# Crystal structure of bis­(mesit­yl)(pyrrol-1-yl)borane

**DOI:** 10.1107/S2056989022011768

**Published:** 2023-01-01

**Authors:** Onur Sahin, John D. Wallis

**Affiliations:** a School of Science and Technology, Nottingham Trent University, Clifton Lane, Nottingham, NG1 8NS, United Kingdom; University of Durham, United Kingdom

**Keywords:** crystal structure, pyrrolo­borane, N-B bonding, π delocalization

## Abstract

In the crystal structure of the title compound, C_22_H_26_BN, the B atom acts to reduce the delocalization of the nitro­gen lone-pair electron density into the pyrrole ring, so that the two N—C bonds increase in length to 1.4005 (14) and 1.3981 (14) Å. The N—B bond length is 1.4425 (15) Å, which is longer than a typical N—B bond because the nitro­gen lone pair is not fully available to participate in the bond.

## Abstract 2

In the crystal structure of the title compound, the dimesitylboron group acts to reduce the delocalization of the nitro­gen atom’s lone pair into the pyrrole ring, with increases in the two N—C bond lengths compared to pyrrole itself. The N—B bond is 1.44125 (15) Å long.

## 1. Chemical context

The structure of the title compound **1** is of inter­est because of the effect of the bis­(mesit­yl)boron group on the electronic structure of the pyrrole, since the nitro­gen lone pair, which is essential to the heterocycle’s aromatic 6π system, now has the possibility of being donated to the boron atom. This mol­ecule has been investigated previously for its fluorescence, and shows a large Stokes shift, which involves twisted intra­molecular charge transfer (TICT) of the excited state, by rotation about the N—B bond (Brittelli & Eaton, 1989 ▸; Cornelissen & Rettig, 1994 ▸; Cornelissen-Gude & Rettig,1999 ▸). It has been used recently in the preparation of conductive polymers (Wildgoose *et al.* 2019 ▸). 
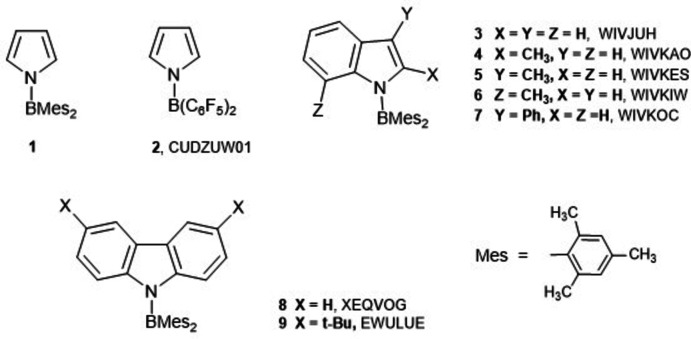


## 2. Structural commentary

**Figure 1:**
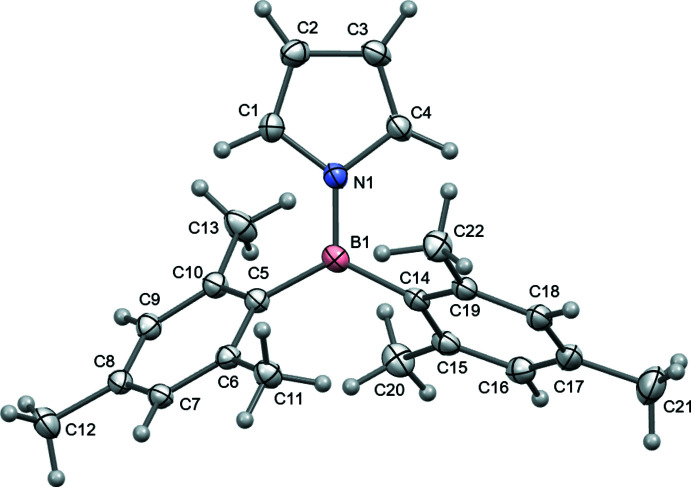
The mol­ecular structure of **1**, with anisotropic displacement parameters drawn at the 50% probability level. Only the more populated orientations for methyl groups C12, C13 and C21 are shown.

The crystal structure of bis­(mesit­yl)(pyrrol-1-yl)borane **1** was determined at 120 K and the mol­ecular structure is shown in Fig. 1 ▸. The bonding geometries at both the boron and nitro­gen atoms are almost planar, with an angle of 16.13 (8)° between these two bonding planes. The N and B atoms lie 0.0351 (11) and 0.0285 (13) Å, respectively, out of the planes defined by their three attached atoms. The planes of the two mesityl groups lie at 58.53 (3) and 61.46 (4)° to the boron atom’s bonding plane, and so there is limited donation of their π-electron densities to boron. These dispositions are controlled by the need to maintain separations between their two adjacent pairs of *ortho* methyl groups [H_3_C⋯CH_3_ = 3.610 (3) and 3.736 (3) Å], and is supported by the widening of the C—B—C bond angle [125.18 (9)°], compared to the two N—B—C bond angles [118.30 (10) and 116.42 (9)°]. The mesityl groups’ planes lie at 77.14 (4)° to each other, and at 69.18 (3) and 67.06 (4)° to the pyrrole ring’s best plane. The hydrogen atoms of three methyl groups (C12, C13 and C21) were modelled in two orientations. The positions and displacement parameters of hydrogen atoms on the pyrrole ring were refined, and for those attached to the C_α_ atoms, the H—C_α_—C_β_ angle showed widening to 131–132°, similar to that in pyrrole (Goddard *et al.*, 1997 ▸; Lee & Boo, 1996 ▸).

The N—B bond is 1.4425 (15) Å long. This is *ca* 0.04 Å longer than in similar compounds where the nitro­gen atom is attached to two *sp* ^3^ carbon atoms [ROCRAD (two mol­ecules; Morawitz *et al.*, 2008 ▸), TAYYAV (Araki *et al.*, 2012 ▸), UWUFID (Smith *et al.*, 2016 ▸), YOMKAM (Khasnis *et al.*, 1995 ▸); *T* ≤ 173 K, N—B range 1.388 (2)-1.412 (3) Å, average 1.40 Å] and where the nitro­gen lone pair is fully available for donation to boron. Compared to the mol­ecular geometry of pyrrole itself, as determined by X-ray crystallography at 103 K (Goddard *et al.*, 1997 ▸) and by calculation at the B3LYP-631G^*^ level (Lee & Boo, 1996 ▸), the most notable difference is in the increase of the two N—C bond lengths to 1.4005 (14) and 1.3981 (14) Å from 1.365 (2) Å (experimental) and 1.376 Å (calculated) (Table 1 ▸). The C_α_—C_β_ bond lengths are 1.3536 (16) and 1.3514 (17) Å and the C_β_—C_β_ bond length is 1.4290 (17) Å. Thus, in contrast to pyrrole, the N—C_α_ and C_α_—C_β_ bonds are no longer similar in length, due to a reduction in the contribution of the nitro­gen atom’s lone pair to the electronic π system of the pyrrole ring. When the mesityl groups are replaced by penta­fluoro­phenyl groups in derivative **2**, the N—B bond is considerably shorter than in **1** [1.4094 (9) *cf*. 1.4425 (15) Å] due to greater lone-pair donation from nitro­gen towards the more electron-deficient boron. Consequently, compared to **1**, the pyrrole ring shows slightly longer N—C bonds [1.4033 (6) Å] and a longer C_β_—C_β_ bond [1.4418 (9) Å], though similar lengths for the C_α_—C_β_ bonds [1.3553 (6) Å] (Table 1 ▸). For comparison, the effect of boron on the pyrrole ring in **1** is similar to that when the pyrrole nitro­gen atom is substituted with a carbonyl group to form an amide (Table 1 ▸).

## 3. Supra­molecular features

**Figure 2:**
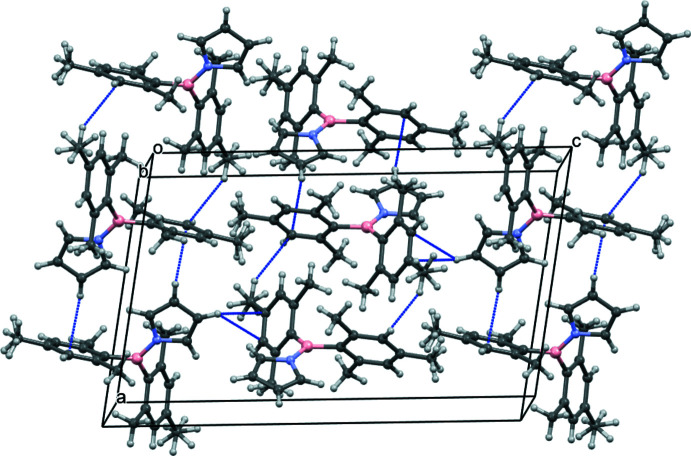
The crystal-packing arrangement for **1**, viewed down the *b* axis, with the shortest C⋯H separations (2.81–2.83 Å) shown in blue. Both orientations of the H atoms on methyl groups C12, C13 and C21 are shown.

The mol­ecules are packed in layers in the *ab* plane (Fig. 2 ▸). There are no particularly short inter­molecular contacts, consistent with the low density of the crystal (1.132 g cm^−3^). Within a layer, the mol­ecules are related by centres of symmetry and translations along *a* and *b*. Adjacent layers are related by the twofold screw and *n*-glide planes. The four shortest inter­molecular C⋯H distances are in the range 2.81–2.83 Å. Two of these involve the *meta*-C atom, C18, with a methyl hydrogen atom and a pyrrole ring’s hydrogen atom, which are directed to opposite sides of the phenyl ring [C18⋯H12*A*(1 − *x*, 1 − *y*, 1 − *z*) and C18⋯H3(−*x*, 2 − *y*, 1 − *z*)]. The two others involve both a *meta* and a *para*-C atom of the second phenyl ring and the pyrrole hydrogen atom H2 [C8⋯H2( 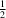 − *x*, *y* − 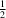 , 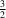 − *z*) and C9⋯H2( 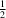 − *x*, *y* − 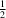 , 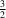 − *z*)].

## 4. Database survey

The structure of the analogue of **1** bearing two 2′-thienyl groups in the pyrrole’s 2- and 5-positions (XEQVUM; Taniguchi *et al.*, 2013 ▸) shows a larger angle (35.1°) between the bonding planes at nitro­gen and boron due to avoidance of steric inter­actions between the thio­phene and mesityl groups, and has a longer N—B bond [1.472 (7) Å] though the structure has lower precision. Room-temperature measurements on a series of five indole analogues of bis­(mesit­yl)pyrrolo­borane, **3**–**7**, show angles of 22.4–32.4° between the two bonding planes and slightly longer N—B bonds [1.442 (3)–1.457 (3) Å] with a correlation between the increasing angle between bonding planes and longer N—B bonds (Cui *et al.*, 2007 ▸). Two carbazole analogues, **8** and **9**, show inter­planar angles between the two bonding planes of 23.1 and 27.9° and N—B bond lengths of 1.442 (3) and 1.440 (3) Å, similar to those in **1** (Taniguchi *et al.*, 2013 ▸; Weber *et al.*, 2011 ▸). All structures are reported in the Cambridge Structural Database, release 2021.3 (Groom *et al.*, 2016 ▸).

## 5. Synthesis and crystallization

A solution of pyrrole (0.25 g, 3.7 mmol) in THF (10 mL) under nitro­gen was treated with sodium hydride (60% dispersion in oil, 0.16 g, 4.0 mmol) and the mixture stirred at 293 K for 2 h. Dimesitylboron fluoride (CARE: gives HF with moisture) (1.09 g, 4.07 mmol) in dry THF (10 mL) was added at room temperature. The mixture was left to stir overnight. The bright orange–yellow mixture was quenched with water (20 mL), extracted with ether (2 × 30 mL) and the combined organic phase was dried over MgSO_4_. The crude material was purified by column chromatography (SiO_2_) using hexa­ne:di­chloro­methane (8:1) as eluent to give **1** (0.76 g, 65%) as a slightly oily white solid, from which crystals were grown using ethyl acetate, m.p. 411 K. ^1^H NMR (400 MHz, CDCl_3_) [ppm]: δ = 6.84 (4H, *s*, 2 × 3′-,5′-*H*), 6.81 (2H, *t*, *J* = 2.2 Hz, 2-,5-*H*), 6.37 (2H, *t*, *J* = 2.2 Hz, 3-,4-*H*), 2.32 (6H, *s*, 2 × 4′-C*H*_3_), 2.11 (12H, *s*, 2 × 2′-, 6′-C*H*_3_); ^13^C NMR (100 MHz, CDCl_3_) [ppm]: δ = 141.7 (2 × 2′-, 6′-*C*), 139.0 (2 × 4′-*C*), 136.5 *br* (2 × 1′-*C*), 128.3 (2 × 3′-, 5′-*C*), 126.5 (2-, 5-*C*), 114.6 (3-, 4-*C*), 22.8 (2 × 2′-, 6′-*C*H_3_), 21.5 (2 × 4′-*C*H_3_); IR (ATR): 2920, 2853, 1606, 1472, 1451, 1421, 1399, 1378, 1329, 1310, 1287, 1252, 1156, 1122, 1080, 1074, 1043, 1030, 850, 817, 763, 733, 717, 677, 656, 619, 560, 516 cm^−1^.

## 6. Refinement

Crystal data and details of data collection and structure refinement are summarized in Table 2 ▸. Pyrrole H-atom positions and displacement parameters were refined. All other H atoms were refined using a riding model with C—H bonds fixed at 0.95 Å for hydrogens attached to phenyl carbon atoms and at 0.98 Å for methyl hydrogen atoms. Three methyl groups were refined in two orientations (C12, C13 and C21). The isotropic atomic displacement parameters of the H atoms were set at 1.2*U*_eq_ of the parent atom for aromatic groups and at 1.5*U*_eq_ for methyl groups.

## Supplementary Material

Crystal structure: contains datablock(s) I. DOI: 10.1107/S2056989022011768/zv2023sup1.cif

Structure factors: contains datablock(s) I. DOI: 10.1107/S2056989022011768/zv2023Isup2.hkl

This is NMR data requested by the referee. DOI: 10.1107/S2056989022011768/zv2023sup3.docx

Supporting information file. DOI: 10.1107/S2056989022011768/zv2023Isup4.cml

CCDC reference: 2166138

Additional supporting information:  crystallographic information; 3D view; checkCIF report

## Figures and Tables

**Table 1:** Bond lengths (Å) for **1** and related compounds

	**1**, *T* = 120 K	Pyrrole,^*a*^ *T* = 103 K	Pyrrole, calculated^*b*^	**2**,^*c*^ *T* = 100 K	Pyrrole, N—C=O^*d*^
N—B	1.4425 (15)	–	–	1.4094 (9)	–
N—C_α_	1.4005 (14) 1.3981 (14)	1.365 (2)	1.376	1.4033 (6)	1.395
C_α_—C_β_	1.3536 (16) 1.3514 (17)	1.357 (2)	1.378	1.3553 (6)	1.355
C_β_—C_β_	1.4290 (17)	1.423 (3)	1.425	1.4418 (9)	1.430

Notes: (*a*) RUVQII (Goddard *et al.*, 1997 ▸); (*b*) Lee & Boo (1996 ▸); (*c*) CUDZUW01 (Flierler *et al.*, 2009 ▸); (*d*) Averaged data for structures containing *C*-unsubstituted-*N*-carbonyl pyrrole fragments measured at *T* < 150 K [BEFFUQ (Hatano *et al.*, 2016 ▸), BOKSUR (Ariyarathna & Tunge, 2014 ▸), CIFNIR (O’Brien *et al.*, 2018 ▸) and LAQFER (Uraguchi *et al.*, 2017 ▸.

**Table 2:** Experimental details

Crystal data	
Chemical formula	C_22_H_26_BN
*M*_r_	315.25
Crystal system, space group	Monoclinic, *P*2_1_/*n*
Temperature (K)	120
*a*, *b*, *c* (Å)	11.9157 (2), 8.0223 (1), 19.6440 (3)
β (°)	99.890 (1)
*V* (Å^3^)	1849.89 (5)
*Z*	4
Radiation type	Cu *K*α
μ (mm^−1^)	0.48
Crystal size (mm)	0.24 × 0.22 × 0.07

Data collection	
Diffractometer	XtaLAB Synergy R, DW system, HyPix-Arc 100
Absorption correction	Gaussian (*CrysAlis PRO*; Rigaku OD, 2022 ▸)
*T*_min_, *T*_max_	0.589, 1.000
No. of measured, independent and observed [*I* > 2σ(*I*)] reflections	12428, 3650, 3305
*R*_int_	0.021
(sin θ/λ)_max_ (Å^−1^)	0.639

Refinement	
*R*[*F* ^2^ > 2σ(*F* ^2^)], *wR*(*F* ^2^), *S*	0.037, 0.102, 1.06
No. of reflections	3650
No. of parameters	240
H-atom treatment	H atoms treated by a mixture of independent and constrained refinement
Δρ_max_, Δρ_min_ (e Å^−3^)	0.28, −0.19

Computer programs: *CrysAlis PRO* (Rigaku OD, 2022 ▸), *SHELXT2018/2* (Sheldrick, 2015*a* ▸), *SHELXL2018/3* (Sheldrick, 2015*b* ▸), *Mercury* (Macrae *et al.*, 2020 ▸) and *OLEX2* (Dolomanov *et al.*, 2009 ▸).

## supplementary crystallographic information

### Crystal data

**Table:**

C_22_H_26_BN	*D*_x_ = 1.132 Mg m^−^^3^
*M**_r_* = 315.25	Melting point: 411 K
Monoclinic, *P*2_1_/*n*	Cu *K*α radiation, λ = 1.54184 Å
*a* = 11.9157 (2) Å	Cell parameters from 8967 reflections
*b* = 8.0223 (1) Å	θ = 4.0–79.1°
*c* = 19.6440 (3) Å	µ = 0.48 mm^−^^1^
β = 99.890 (1)°	*T* = 120 K
*V* = 1849.89 (5) Å^3^	Plate, colourless
*Z* = 4	0.24 × 0.22 × 0.07 mm
*F*(000) = 680	

### Data collection

**Table:**

XtaLAB Synergy R, DW system, HyPix-Arc 100 diffractometer	3650 independent reflections
Radiation source: Rotating-anode X-ray tube, Rigaku (Cu) X-ray Source	3305 reflections with *I* > 2σ(*I*)
Mirror monochromator	*R*_int_ = 0.021
Detector resolution: 10.0000 pixels mm^-1^	θ_max_ = 80.4°, θ_min_ = 4.1°
ω scans	*h* = −15→14
Absorption correction: gaussian (CrysAlisPro; Rigaku OD, 2022)	*k* = −3→10
*T*_min_ = 0.589, *T*_max_ = 1.000	*l* = −23→24
12428 measured reflections	

### Refinement

**Table:**

Refinement on *F*^2^	Hydrogen site location: mixed
Least-squares matrix: full	H atoms treated by a mixture of independent and constrained refinement
*R*[*F*^2^ > 2σ(*F*^2^)] = 0.037	*w* = 1/[σ^2^(*F*_o_^2^) + (0.0503*P*)^2^ + 0.5405*P*] where *P* = (*F*_o_^2^ + 2*F*_c_^2^)/3
*wR*(*F*^2^) = 0.102	(Δ/σ)_max_ = 0.001
*S* = 1.06	Δρ_max_ = 0.28 e Å^−^^3^
3650 reflections	Δρ_min_ = −0.19 e Å^−^^3^
240 parameters	Extinction correction: *SHELXL2018/3* (Sheldrick, 2015b), Fc^*^=kFc[1+0.001xFc^2^λ^3^/sin(2θ)]^-1/4^
0 restraints	Extinction coefficient: 0.0035 (4)
Primary atom site location: dual	

### Special details

**Table:**

Geometry. All esds (except the esd in the dihedral angle between two l.s. planes) are estimated using the full covariance matrix. The cell esds are taken into account individually in the estimation of esds in distances, angles and torsion angles; correlations between esds in cell parameters are only used when they are defined by crystal symmetry. An approximate (isotropic) treatment of cell esds is used for estimating esds involving l.s. planes.

### Fractional atomic coordinates and isotropic or equivalent isotropic displacement parameters (Å^2^)

**Table:**

	*x*	*y*	*z*	*U*_iso_*/*U*_eq_	Occ. (<1)
N1	0.18097 (7)	0.77715 (11)	0.59306 (5)	0.0222 (2)	
C1	0.18799 (10)	0.78480 (14)	0.66489 (6)	0.0252 (3)	
H1	0.2424 (11)	0.7174 (17)	0.6936 (7)	0.027 (3)*	
C2	0.10761 (10)	0.89098 (15)	0.68019 (6)	0.0276 (3)	
H2	0.0938 (12)	0.9190 (18)	0.7256 (8)	0.034 (4)*	
C3	0.04576 (10)	0.95241 (15)	0.61633 (6)	0.0263 (3)	
H3	−0.0173 (12)	1.0297 (19)	0.6110 (7)	0.034 (4)*	
C4	0.09209 (9)	0.88346 (14)	0.56483 (6)	0.0243 (2)	
H4	0.0713 (11)	0.8919 (17)	0.5145 (7)	0.028 (3)*	
C5	0.31151 (9)	0.51849 (13)	0.59337 (5)	0.0203 (2)	
C6	0.43044 (9)	0.49950 (13)	0.59754 (5)	0.0204 (2)	
C7	0.48525 (9)	0.35880 (14)	0.62875 (5)	0.0220 (2)	
H7	0.565353	0.348470	0.631666	0.026*	
C8	0.42558 (9)	0.23297 (14)	0.65577 (5)	0.0225 (2)	
C9	0.30873 (9)	0.25156 (14)	0.65143 (5)	0.0224 (2)	
H9	0.266773	0.165907	0.669121	0.027*	
C10	0.25120 (9)	0.39203 (14)	0.62194 (6)	0.0222 (2)	
C11	0.50069 (9)	0.63324 (14)	0.57070 (6)	0.0250 (3)	
H11A	0.465994	0.662557	0.523314	0.038*	
H11B	0.578176	0.591750	0.571152	0.038*	
H11C	0.503495	0.732173	0.600204	0.038*	
C12	0.48501 (11)	0.08266 (16)	0.69069 (7)	0.0324 (3)	
H12A	0.566645	0.089460	0.689114	0.039*	0.459 (14)
H12B	0.473774	0.079013	0.738926	0.039*	0.459 (14)
H12C	0.453368	−0.018501	0.666784	0.039*	0.459 (14)
H12D	0.429213	0.010521	0.707435	0.039*	0.541 (14)
H12E	0.522084	0.020969	0.657624	0.039*	0.541 (14)
H12F	0.542491	0.118483	0.729766	0.039*	0.541 (14)
C13	0.12378 (10)	0.39744 (16)	0.62051 (7)	0.0318 (3)	
H13A	0.092836	0.501380	0.598706	0.038*	0.663 (14)
H13B	0.087803	0.302290	0.594031	0.038*	0.663 (14)
H13C	0.108254	0.392161	0.667858	0.038*	0.663 (14)
H13D	0.099760	0.295840	0.641691	0.038*	0.337 (14)
H13E	0.104792	0.494931	0.646366	0.038*	0.337 (14)
H13F	0.084341	0.405060	0.572538	0.038*	0.337 (14)
C14	0.23755 (8)	0.71202 (13)	0.47517 (5)	0.0203 (2)	
C15	0.19186 (9)	0.59532 (14)	0.42397 (6)	0.0227 (2)	
C16	0.17238 (9)	0.64178 (15)	0.35462 (6)	0.0267 (3)	
H16	0.139938	0.562717	0.320900	0.032*	
C17	0.19871 (9)	0.79950 (16)	0.33310 (6)	0.0271 (3)	
C18	0.24852 (9)	0.91129 (15)	0.38323 (6)	0.0245 (2)	
H18	0.269808	1.018547	0.369374	0.029*	
C19	0.26830 (9)	0.87098 (14)	0.45335 (6)	0.0217 (2)	
C20	0.16008 (10)	0.42065 (15)	0.44205 (7)	0.0310 (3)	
H20A	0.226974	0.364914	0.468333	0.046*	
H20B	0.133123	0.358118	0.399531	0.046*	
H20C	0.099533	0.425544	0.470029	0.046*	
C21	0.17317 (13)	0.8475 (2)	0.25774 (7)	0.0422 (3)	
H21A	0.197459	0.962807	0.252349	0.051*	0.603 (17)
H21B	0.214358	0.773065	0.231077	0.051*	0.603 (17)
H21C	0.091124	0.837973	0.240847	0.051*	0.603 (17)
H21D	0.137835	0.753090	0.230500	0.051*	0.397 (17)
H21E	0.120936	0.942831	0.251772	0.051*	0.397 (17)
H21F	0.244170	0.877924	0.242002	0.051*	0.397 (17)
C22	0.32315 (11)	1.00106 (15)	0.50381 (6)	0.0291 (3)	
H22A	0.263881	1.069083	0.519144	0.044*	
H22B	0.372504	1.072516	0.481251	0.044*	
H22C	0.368705	0.946066	0.543823	0.044*	
B1	0.24540 (10)	0.67087 (15)	0.55475 (6)	0.0209 (3)	

### Atomic displacement parameters (Å^2^)

**Table:**

	*U*^11^	*U*^22^	*U*^33^	*U*^12^	*U*^13^	*U*^23^
N1	0.0223 (4)	0.0212 (5)	0.0232 (5)	0.0039 (4)	0.0045 (4)	0.0042 (4)
C1	0.0285 (6)	0.0243 (6)	0.0233 (5)	0.0026 (5)	0.0056 (4)	0.0045 (5)
C2	0.0328 (6)	0.0251 (6)	0.0275 (6)	0.0012 (5)	0.0124 (5)	0.0024 (5)
C3	0.0235 (5)	0.0232 (6)	0.0339 (6)	0.0043 (5)	0.0099 (5)	0.0035 (5)
C4	0.0225 (5)	0.0227 (6)	0.0276 (6)	0.0044 (4)	0.0042 (4)	0.0047 (4)
C5	0.0206 (5)	0.0198 (5)	0.0202 (5)	0.0023 (4)	0.0028 (4)	0.0000 (4)
C6	0.0207 (5)	0.0217 (5)	0.0186 (5)	0.0015 (4)	0.0031 (4)	−0.0026 (4)
C7	0.0194 (5)	0.0251 (6)	0.0209 (5)	0.0043 (4)	0.0022 (4)	−0.0022 (4)
C8	0.0267 (5)	0.0211 (5)	0.0190 (5)	0.0054 (4)	0.0018 (4)	−0.0010 (4)
C9	0.0263 (5)	0.0194 (5)	0.0216 (5)	−0.0002 (4)	0.0047 (4)	0.0007 (4)
C10	0.0214 (5)	0.0222 (5)	0.0230 (5)	0.0018 (4)	0.0039 (4)	−0.0003 (4)
C11	0.0207 (5)	0.0253 (6)	0.0292 (6)	0.0003 (4)	0.0046 (4)	0.0016 (5)
C12	0.0328 (6)	0.0283 (6)	0.0355 (7)	0.0095 (5)	0.0040 (5)	0.0069 (5)
C13	0.0224 (5)	0.0272 (6)	0.0467 (7)	0.0020 (5)	0.0087 (5)	0.0082 (5)
C14	0.0166 (5)	0.0205 (5)	0.0235 (5)	0.0045 (4)	0.0028 (4)	0.0007 (4)
C15	0.0164 (5)	0.0242 (6)	0.0270 (6)	0.0034 (4)	0.0020 (4)	−0.0024 (4)
C16	0.0204 (5)	0.0332 (6)	0.0255 (6)	0.0020 (5)	0.0014 (4)	−0.0063 (5)
C17	0.0206 (5)	0.0383 (7)	0.0226 (5)	0.0058 (5)	0.0043 (4)	0.0017 (5)
C18	0.0223 (5)	0.0259 (6)	0.0265 (6)	0.0047 (4)	0.0073 (4)	0.0049 (5)
C19	0.0197 (5)	0.0215 (5)	0.0245 (5)	0.0044 (4)	0.0051 (4)	0.0008 (4)
C20	0.0297 (6)	0.0246 (6)	0.0361 (7)	−0.0027 (5)	−0.0015 (5)	−0.0027 (5)
C21	0.0445 (7)	0.0562 (9)	0.0246 (6)	0.0007 (7)	0.0026 (5)	0.0051 (6)
C22	0.0364 (6)	0.0214 (6)	0.0295 (6)	−0.0021 (5)	0.0057 (5)	0.0009 (5)
B1	0.0177 (5)	0.0188 (6)	0.0256 (6)	−0.0012 (4)	0.0023 (4)	0.0007 (5)

### Geometric parameters (Å, º)

**Table:**

N1—C1	1.4005 (14)	C13—H13A	0.9800
N1—C4	1.3981 (14)	C13—H13B	0.9800
N1—B1	1.4425 (15)	C13—H13C	0.9800
C1—H1	0.951 (14)	C13—H13D	0.9800
C1—C2	1.3536 (16)	C13—H13E	0.9800
C2—H2	0.962 (15)	C13—H13F	0.9800
C2—C3	1.4290 (17)	C14—C15	1.4129 (15)
C3—H3	0.966 (15)	C14—C19	1.4135 (15)
C3—C4	1.3514 (17)	C14—B1	1.5848 (16)
C4—H4	0.979 (14)	C15—C16	1.3928 (16)
C5—C6	1.4135 (14)	C15—C20	1.5097 (16)
C5—C10	1.4139 (15)	C16—H16	0.9500
C5—B1	1.5765 (15)	C16—C17	1.3870 (18)
C6—C7	1.3924 (15)	C17—C18	1.3877 (17)
C6—C11	1.5106 (15)	C17—C21	1.5093 (16)
C7—H7	0.9500	C18—H18	0.9500
C7—C8	1.3913 (16)	C18—C19	1.3950 (15)
C8—C9	1.3883 (15)	C19—C22	1.5087 (16)
C8—C12	1.5030 (15)	C20—H20A	0.9800
C9—H9	0.9500	C20—H20B	0.9800
C9—C10	1.3925 (15)	C20—H20C	0.9800
C10—C13	1.5144 (15)	C21—H21A	0.9800
C11—H11A	0.9800	C21—H21B	0.9800
C11—H11B	0.9800	C21—H21C	0.9800
C11—H11C	0.9800	C21—H21D	0.9800
C12—H12A	0.9800	C21—H21E	0.9800
C12—H12B	0.9800	C21—H21F	0.9800
C12—H12C	0.9800	C22—H22A	0.9800
C12—H12D	0.9800	C22—H22B	0.9800
C12—H12E	0.9800	C22—H22C	0.9800
C12—H12F	0.9800		
			
C1—N1—B1	127.34 (9)	C10—C13—H13C	109.5
C4—N1—C1	106.43 (9)	H13A—C13—H13B	109.5
C4—N1—B1	126.05 (9)	H13A—C13—H13C	109.5
N1—C1—H1	119.3 (8)	H13B—C13—H13C	109.5
C2—C1—N1	109.23 (10)	H13D—C13—H13E	109.5
C2—C1—H1	131.5 (8)	H13D—C13—H13F	109.5
C1—C2—H2	126.4 (9)	H13E—C13—H13F	109.5
C1—C2—C3	107.45 (10)	C15—C14—C19	118.05 (10)
C3—C2—H2	126.1 (9)	C15—C14—B1	120.96 (10)
C2—C3—H3	126.2 (8)	C19—C14—B1	120.83 (10)
C4—C3—C2	107.50 (10)	C14—C15—C20	121.94 (10)
C4—C3—H3	126.3 (8)	C16—C15—C14	119.87 (11)
N1—C4—H4	119.1 (8)	C16—C15—C20	118.17 (10)
C3—C4—N1	109.38 (10)	C15—C16—H16	118.9
C3—C4—H4	131.4 (8)	C17—C16—C15	122.27 (11)
C6—C5—C10	118.19 (9)	C17—C16—H16	118.9
C6—C5—B1	121.64 (9)	C16—C17—C18	117.70 (10)
C10—C5—B1	120.09 (9)	C16—C17—C21	120.93 (12)
C5—C6—C11	120.89 (9)	C18—C17—C21	121.38 (12)
C7—C6—C5	120.17 (10)	C17—C18—H18	119.0
C7—C6—C11	118.90 (9)	C17—C18—C19	122.01 (11)
C6—C7—H7	119.2	C19—C18—H18	119.0
C8—C7—C6	121.62 (10)	C14—C19—C22	122.03 (10)
C8—C7—H7	119.2	C18—C19—C14	119.99 (10)
C7—C8—C12	121.64 (10)	C18—C19—C22	117.98 (10)
C9—C8—C7	118.13 (10)	C15—C20—H20A	109.5
C9—C8—C12	120.21 (10)	C15—C20—H20B	109.5
C8—C9—H9	119.0	C15—C20—H20C	109.5
C8—C9—C10	121.96 (10)	H20A—C20—H20B	109.5
C10—C9—H9	119.0	H20A—C20—H20C	109.5
C5—C10—C13	123.26 (10)	H20B—C20—H20C	109.5
C9—C10—C5	119.89 (10)	C17—C21—H21A	109.5
C9—C10—C13	116.82 (10)	C17—C21—H21B	109.5
C6—C11—H11A	109.5	C17—C21—H21C	109.5
C6—C11—H11B	109.5	H21A—C21—H21B	109.5
C6—C11—H11C	109.5	H21A—C21—H21C	109.5
H11A—C11—H11B	109.5	H21B—C21—H21C	109.5
H11A—C11—H11C	109.5	H21D—C21—H21E	109.5
H11B—C11—H11C	109.5	H21D—C21—H21F	109.5
C8—C12—H12A	109.5	H21E—C21—H21F	109.5
C8—C12—H12B	109.5	C19—C22—H22A	109.5
C8—C12—H12C	109.5	C19—C22—H22B	109.5
H12A—C12—H12B	109.5	C19—C22—H22C	109.5
H12A—C12—H12C	109.5	H22A—C22—H22B	109.5
H12B—C12—H12C	109.5	H22A—C22—H22C	109.5
H12D—C12—H12E	109.5	H22B—C22—H22C	109.5
H12D—C12—H12F	109.5	N1—B1—C5	118.30 (10)
H12E—C12—H12F	109.5	N1—B1—C14	116.42 (9)
C10—C13—H13A	109.5	C5—B1—C14	125.18 (9)
C10—C13—H13B	109.5		
			
N1—C1—C2—C3	0.71 (13)	C15—C14—C19—C18	2.85 (15)
C1—N1—C4—C3	−0.47 (13)	C15—C14—C19—C22	−176.89 (10)
C1—N1—B1—C5	14.84 (16)	C15—C14—B1—N1	−117.91 (11)
C1—N1—B1—C14	−168.66 (10)	C15—C14—B1—C5	58.32 (14)
C1—C2—C3—C4	−1.00 (14)	C15—C16—C17—C18	1.52 (16)
C2—C3—C4—N1	0.90 (13)	C15—C16—C17—C21	−178.10 (11)
C4—N1—C1—C2	−0.17 (13)	C16—C17—C18—C19	−2.32 (16)
C4—N1—B1—C5	−159.49 (10)	C17—C18—C19—C14	0.13 (16)
C4—N1—B1—C14	17.01 (16)	C17—C18—C19—C22	179.88 (10)
C5—C6—C7—C8	0.76 (16)	C19—C14—C15—C16	−3.63 (15)
C6—C5—C10—C9	−1.72 (15)	C19—C14—C15—C20	177.97 (10)
C6—C5—C10—C13	−179.75 (10)	C19—C14—B1—N1	57.41 (13)
C6—C5—B1—N1	−122.50 (11)	C19—C14—B1—C5	−126.35 (11)
C6—C5—B1—C14	61.33 (15)	C20—C15—C16—C17	179.94 (10)
C6—C7—C8—C9	−0.56 (16)	C21—C17—C18—C19	177.29 (11)
C6—C7—C8—C12	−178.79 (10)	B1—N1—C1—C2	−175.40 (11)
C7—C8—C9—C10	−0.83 (16)	B1—N1—C4—C3	174.83 (10)
C8—C9—C10—C5	1.98 (16)	B1—C5—C6—C7	−176.49 (10)
C8—C9—C10—C13	−179.85 (10)	B1—C5—C6—C11	5.66 (15)
C10—C5—C6—C7	0.38 (15)	B1—C5—C10—C9	175.21 (10)
C10—C5—C6—C11	−177.47 (10)	B1—C5—C10—C13	−2.83 (16)
C10—C5—B1—N1	60.69 (14)	B1—C14—C15—C16	171.82 (9)
C10—C5—B1—C14	−115.48 (12)	B1—C14—C15—C20	−6.58 (15)
C11—C6—C7—C8	178.65 (10)	B1—C14—C19—C18	−172.60 (9)
C12—C8—C9—C10	177.43 (10)	B1—C14—C19—C22	7.66 (15)
C14—C15—C16—C17	1.48 (16)		
