# Supplementary figures and images for: Crystal structure of bis­(mesit­yl)(pyrrol-1-yl)borane
Source: Acta Crystallogr E Crystallogr Commun. 2023 Jan 1;79(Pt 1):50–3. doi: 10.1107/S2056989022011768 (PMC9815137; doi:10.1107/S2056989022011768)

**1H NMR Spectrum of Compound 1.**


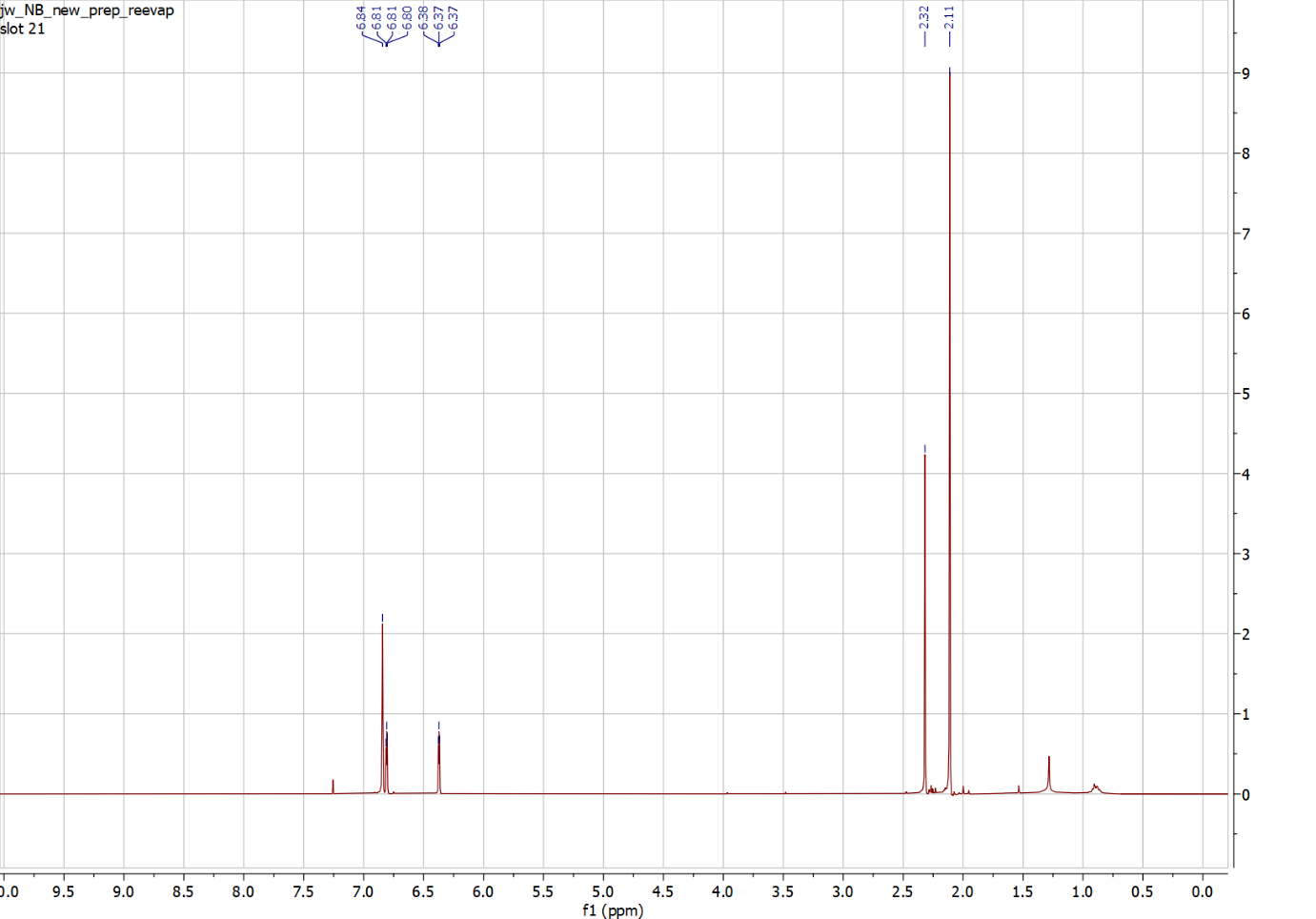


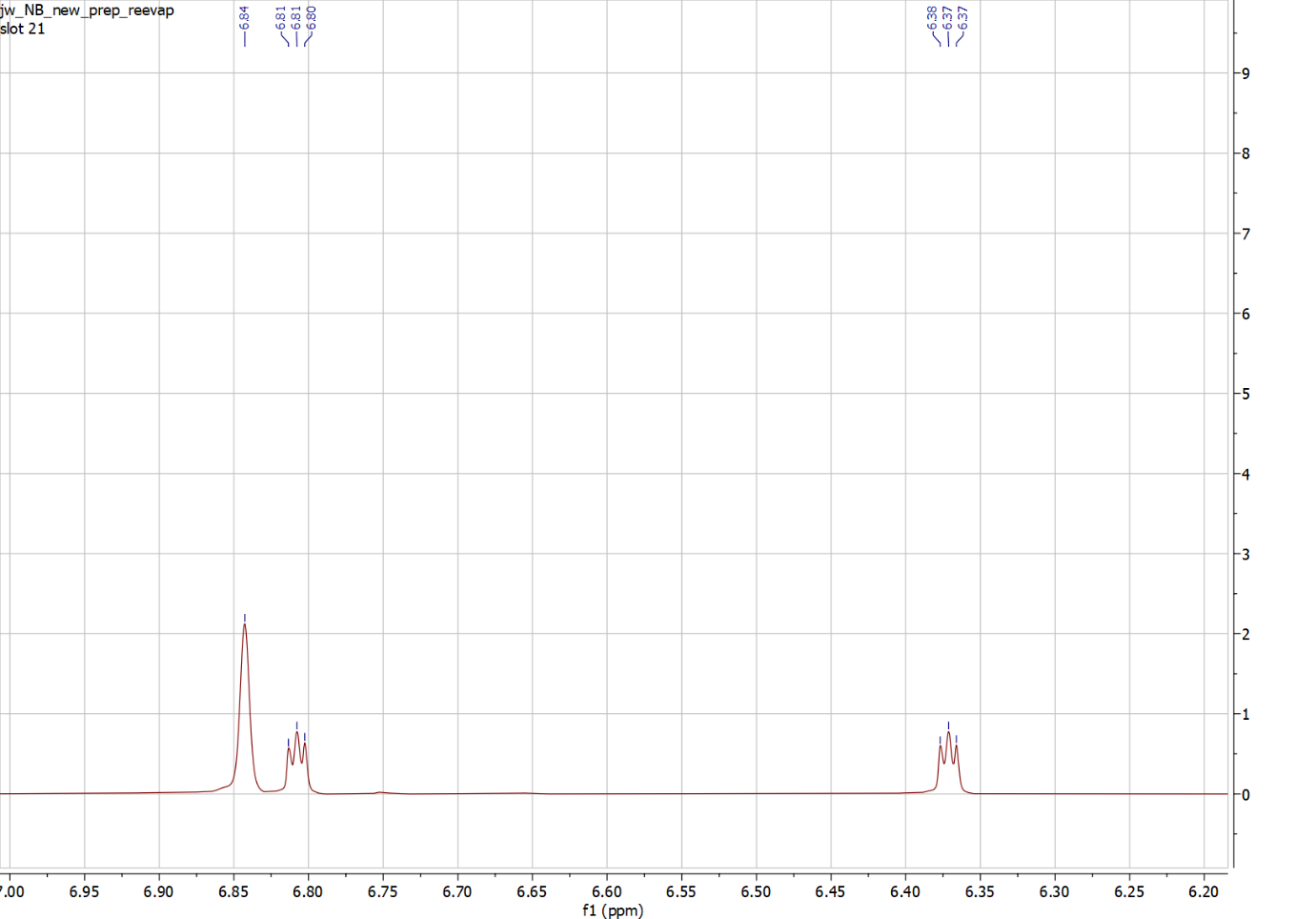


**13C NMR Spectrum of Compound 1.**


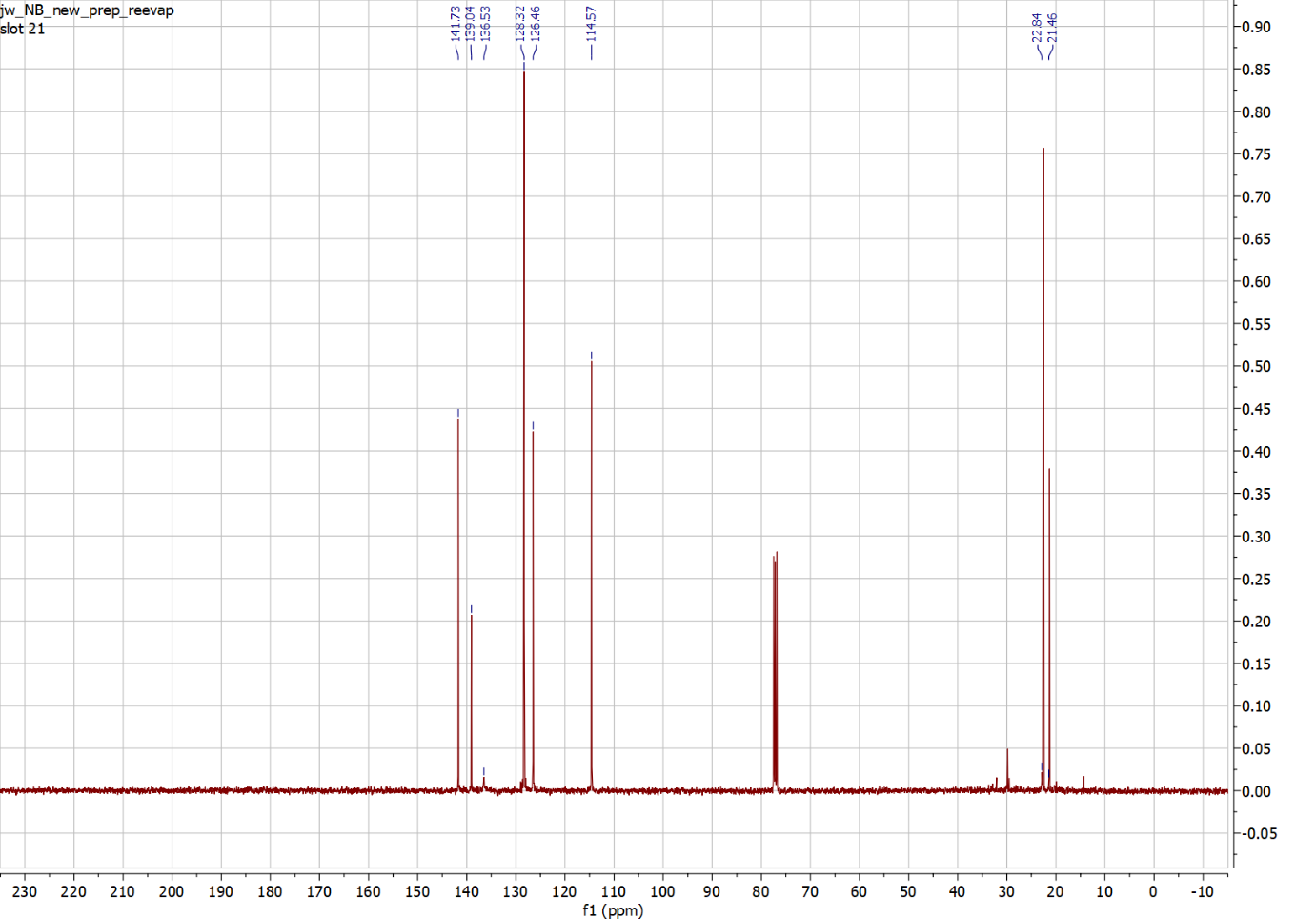


Spectra published using MestreNova.

Supplement: Supplementary file 3 [file e-79-00050-sup3.docx]
